# *Aedes aegypti* microRNA miR-2b regulates ubiquitin-related modifier to control chikungunya virus replication

**DOI:** 10.1038/s41598-017-18043-0

**Published:** 2017-12-15

**Authors:** Sunil Kumar Dubey, Jatin Shrinet, Jaspreet Jain, Shakir Ali, Sujatha Sunil

**Affiliations:** 1 Vector Borne Disease Group, International Centre for Genetic Engineering and Biotechnology (ICGEB), Aruna Asaf Ali Marg, New Delhi, India; 2 Department of Biochemistry, Faculty of Science, Jamia Hamdard, New Delhi, India

## Abstract

Arboviruses that replicate in mosquitoes activate innate immune response within mosquitoes. Regulatory non-coding microRNAs (miRNA) are known to be modulated in mosquitoes during chikungunya infection. However, information about targets of these miRNAs is scant. The present study was aimed to identify and analyze targets of miRNAs that are regulated during chikungunya virus (CHIKV) replication in *Aedes aegypti* cells and in the mosquito. Employing next-generation sequencing technologies, we identified a total of 126 miRNAs from the *Ae. aegypti* cell line Aag2. Of these, 13 miRNAs were found to be regulated during CHIKV infection. Putative targets of three of the most significantly regulated miRNAs- miR-100, miR-2b and miR-989 were also analyzed using quantitative PCRs, in cell lines and in mosquitoes, to validate whether they were the targets of the miRNAs. Our study expanded the list of miRNAs known in *Ae. aegypti* and predicted targets for the significantly regulated miRNAs. Further analysis of some of these targets revealed that ubiquitin-related modifier is a target of miRNA miR-2b and plays a significant role in chikungunya replication.

## Introduction

Innate immunity in insects has been described as the germ line-encoded anti-infection response of the host^1^. The response is executed via several mechanisms such as phagocytosis^2^, antimicrobial peptides^3^, melanotic encapsulation^4^, and nitrogen intermediates^5^. In addition to these mechanisms, insects employ other pathways such as RNA interference (RNAi), immune deficiency (IMD), and toll and JAK/STAT signaling pathways to provide defense against pathogens, including viruses^6,7^. Amongst viruses, some are transmitted by arthropods and are called arboviruses, many of which are of importance due to the impact they have on human health^8^. Mostly transmitted by the *Ae. aegypti* and *Culex tritaeniorhynchus* mosquitoes, these viruses are characterized by their requirement to alternate between a vertebrate host and the mosquito.

Chikungunya virus (CHIKV) is an arbovirus belonging to the genus *Alphavirus*, family *Togaviridae*. Its genome is a single-stranded positive-sense RNA that transcribes mRNA from two open reading frames (ORFs): one that expresses nonstructural proteins and another that expresses structural proteins. In addition to these two ORFs, the genome contains a 5′-terminal cap and a 3′-terminal poly(A) tract along with 5′ and 3′ untranslated regions (UTRs) that contain signals important for replication of the RNA^9^. Whereas much information is available with respect to the innate and adaptive immune response of CHIKV in the mammalian hosts^10,11^, little is known about the innate immune responses in the vector during CHIKV replication^12^. Amongst all innate immune responses, RNAi assumes the first level of defense against RNA viruses in mosquitoes.

RNAi is a conserved, sequence-specific, gene-silencing phenomenon that is induced by double-stranded RNA. In the case of a viral infection in insects, viral replication intermediates in the form of double-stranded RNA (dsRNA) trigger RNAi that acts as a defense mechanism^13^. Whereas exogenous RNAi is triggered by the presence of siRNA generated through the processing of dsRNA and virus-derived siRNAs (vsiRNAs), another class of small RNAs that are regulatory are the microRNAs (miRNA)^14^. A class of small, non-coding RNAs of 19–24 nt in length, miRNAs regulate gene expression post-transcriptionally by binding to complementary regions in the 3′ UTR of target mRNAs^15^. Reports have revealed the role of miRNAs in several cellular processes^16^, including regulating virus replication^17^. However, studies on the cellular targets through which these miRNAs may act are scant, thereby not providing enough information on the mode of action of miRNAs in insect immunity.

A recent study by our group revealed that CHIKV replication in *Aedes albopictus* cells regulated vector miRNAs^18^. The present study was initiated to study the impact of the cellular targets of some of the miRNAs that are regulated in *Ae. aegypti*. Upon CHIKV infection in an *Ae. aegypti* cell line, namely Aag2, significantly regulated miRNAs were identified through next-generation sequencing, targets of the significantly regulated miRNAs were predicted through computational approaches, and pathway analysis of these targets were performed. Furthermore, through loss-of-function assays, targets of the selected miRNAs were validated using quantitative PCR, both in *Ae. aegypti* cells and in *Ae. aegypti* mosquitoes. The present study also showed that miR-2b targets the 3′UTR of ubiquitin-related modifier (URM) to control CHIKV replication.

## Results

Global miRNA profiling of *Ae. aegypti* was performed using high-throughput small RNA sequencing. Small RNA libraries obtained from a CHIKV-infected *Ae. aegypti* cell line (Aag2) at 12 and 24 hours post-infection (h.p.i.) were compared with *Ae. aegypti* miRNAs from an uninfected Aag2 cell line. These time points were chosen in order to evaluate the vector miRNAs during early-phase CHIKV infection. Post-sequencing, small RNA reads from all the three libraries were separately mapped on the *Ae. aegypti* genome. The mapping showed 76.12% reads of uninfected library, 82.34% reads of 12 h.p.i. library, and 51.63% reads of 24 h.p.i. library. The mapped reads were further used for the identification of known miRNAs, including up to one mismatch using data analysis pipeline established in-house^18^. Unmapped reads of 12 and 24 h.p.i. libraries were further mapped on to the CHIKV genome. Details of small RNA analysis of the datasets are shown in Supplementary Tables S2 and S3.

### *Ae. aegypti* miRNAs are regulated upon CHIKV infection

**Figure 1:**
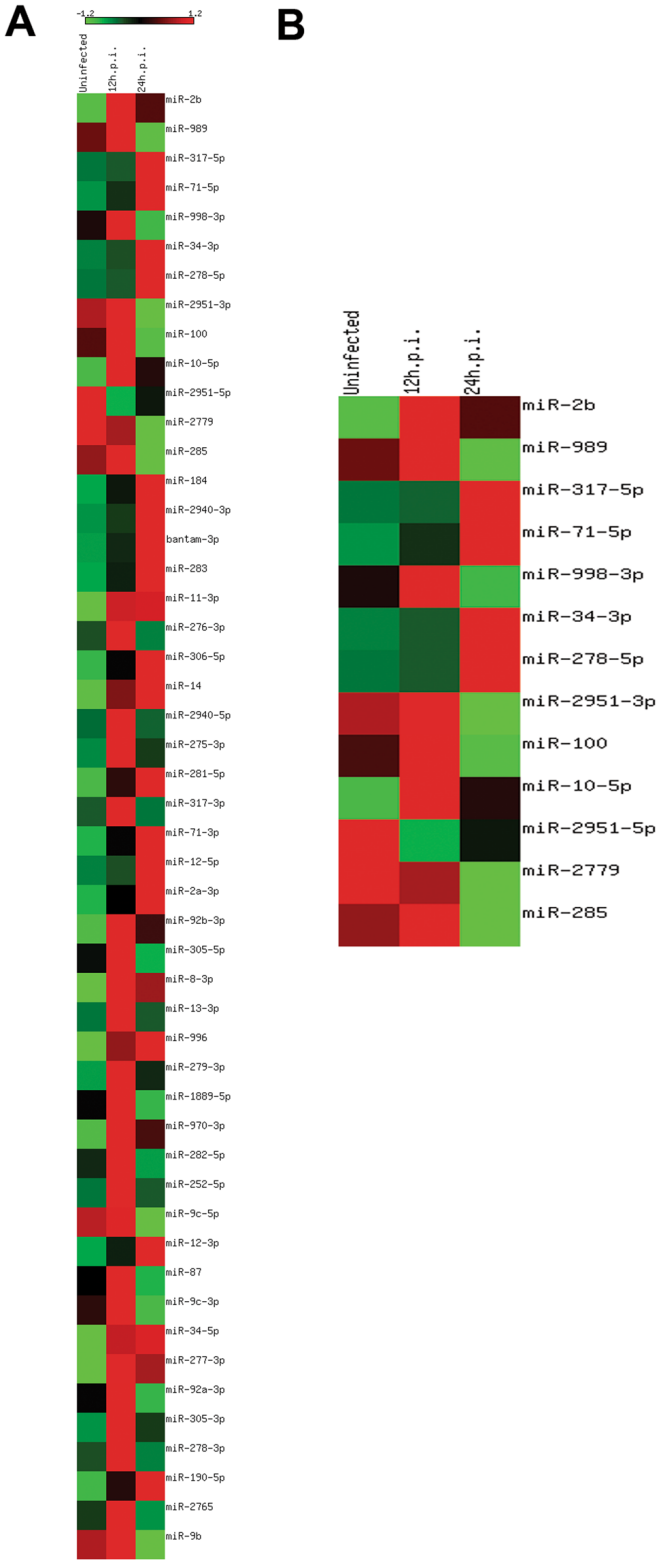
Heatmap of regulated (*Ae. aegypti* cell line) Aag2 miRNAs upon different time points of CHIKV infection. (**A**) Heatmap of top 50 regulated miRNAs. (**B**) Heatmap of significantly regulated miRNAs.

To analyze the relative abundance and expression profiling of miRNAs, tags per million (TPM) of total RNA reads for each miRNA in all libraries were calculated. The TPM values were compared between libraries and a heat map of all predicted miRNAs was generated and visualized. We normalized the row values to have mean of 0 and a variance of 1 **(**Fig. 1A **)**. Log-fold change and *p*-value of the predicted miRNAs were calculated using edgeR packages (trimmed mean of M-values; TMM) described elsewhere^18^. miRNAs with *p*-value ≤ 0.05 were considered significant. The analysis revealed a total of thirteen miRNAs to be significant in all analyses **(**Fig. 1B **)**. Three miRNAs (miR-2b, miR-2951–5p, and miR-10–5p) were found to be significantly regulated (*p* ≤ 0.05) in the 12 h.p.i. library when compared with the uninfected library: two miRNAs (miR-10-5p and miR-2b) were upregulated, and one miRNA (miR-2951-5p) was found to be downregulated. Similarly, eleven miRNAs were significantly regulated in the 24 h.p.i. library, with five miRNAs (miR-34-3p, miR-317-5p, miR-278-5p, miR-2b, and miR-71-5p) showing overexpression and six miRNAs (miR-998-3p, miR-989, miR-285, miR-2779, miR-2951-3p, and miR-100) being underexpressed.

### Validation of miRNA expression using quantitative real-time PCR

**Figure 2:**
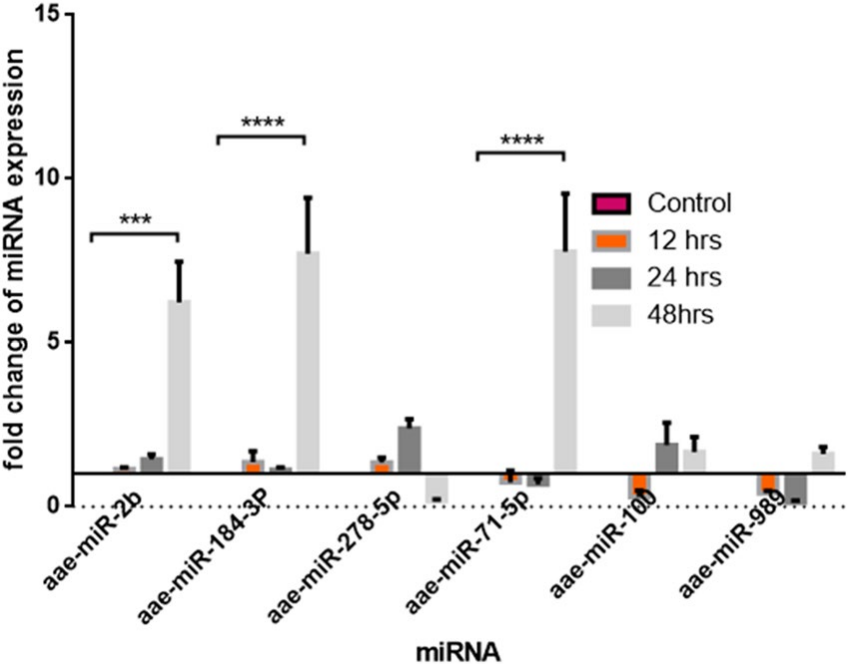
qRT-PCR analysis of selected six miRNAs showing differential regulation upon CHIKV infection at 12, 24, and 48 h, validating deep sequencing results. Data were expressed as mean ± SEM; ****p < 0.0001 *vs*. control group.

To validate the small RNA sequencing results, we carried out quantitative real-time PCR (qRT-PCR) analysis for randomly picked six miRNAs. Four out of the six miRNAs showed expression profiles similar to those observed in small RNA sequencing analysis, whereas miR-100 and miR-71-5p showed different patterns. Quantitative RT-PCR showed that at 24 h.p.i., miR-989 and miR-71-5p were downregulated and miR-2b, miR-184-3p, and miR-278-5p along with miR-2b were upregulated. This scenario changed at 48 h.p.i. in the case of miR-2b, miR-184-3p, and miR-71-5p: these miRNAs showed significant upregulation in their expression at 48 h.p.i. miR-2b showed a six-fold increase (*p* < 0.001), miR-184-3p showed a 7.5-fold increase (*p* < 0.0001), miR-71-5p showed a 7.7-fold increase (*p* < 0.0001), whereas changes in the expression profiles of miR-278-5p, miR-989, and miR-100 remained statistically insignificant **(**Fig. 2). With respect to the two miRNAs that showed a different expression pattern from small RNA sequencing, namely, miR-71-5p and miR-100, we sought to understand the discrepancy in the results between the two techniques. In the case of miR-71-5p, sequencing data analysis showed TPM values to be 15.63, 23.93, and 58.85 for the uninfected, 12 h.p.i. and 24 h.p.i. libraries, respectively. It should be noted that even though the regulation was statistically significant in small RNA data analysis, expression of this miRNA might be too low, which could be a reason for the insignificant qRT-PCR expression profiling even at 24 h. With respect to miR-100, the TPM in the library were higher: 172.42, 288.68, and 9.81 in the uninfected, 12 h.p.i., and 24 h.p.i. library, respectively. However, this pattern was reversed in qPCR analysis, which warrants further studies. Earlier studies reported that such discrepancies are possible due to the inherent probing methods of the technologies^19–21^. All RT-PCR-amplified products were cloned and sequenced to validate the amplification of correct miRNAs. BLAST results revealed specific miRNAs amplified by RT-PCR, thereby further validating small RNA sequencing analysis.

### Target prediction of significant miRNAs and functional analysis

To understand the function of miRNAs, it is important to study their targets. miRNAs are known to function in clusters to regulate various biological processes^22,23^, which prompted us to predict the targets of all the differentially regulated miRNAs that were regulated under different experimental conditions. Targets with the minimum free energy not exceeding −20 kcal/mol and *p* ≤ 0.05 were considered significant and were further subjected to pathway enrichment analysis **(**Supplementary Figure S1 **)**.

### Expression profiling of targets upon miRNA inhibition in *Ae. aegypti* cell lines and mosquitoes

In order to confirm the targets that were predicted, we selected three miRNAs, namely, miR-2b, miR-100, and miR-989 for target prediction, that were among the most significantly differentially expressed in small RNA sequencing, further downstream validations and biological relevance in other systems. A total of 785, 1024, and 294 targets were predicted for miR-2b, miR-989, and miR-100, respectively. Further analysis was performed on the basis of the involvement of targets in immune and signaling pathways, and their binding energies and two targets for each of the three miRNAs were validated further. For miR-2b, two genes, namely, URM (AAEL008680) and ubiquitin (AAEL006511), were selected on the basis of statistical significance and their relevance in immune-related and signaling pathways. In the case of miR-100, cdc42 (AAEL011500) and sumo-ligase (AAEL015099) were selected. Similarly, for miR-989, sh2/sh3 adaptor (AAEL013539) and vacuolar ATP synthase (AAEL012819) were selected for validation.

**Figure 3:**
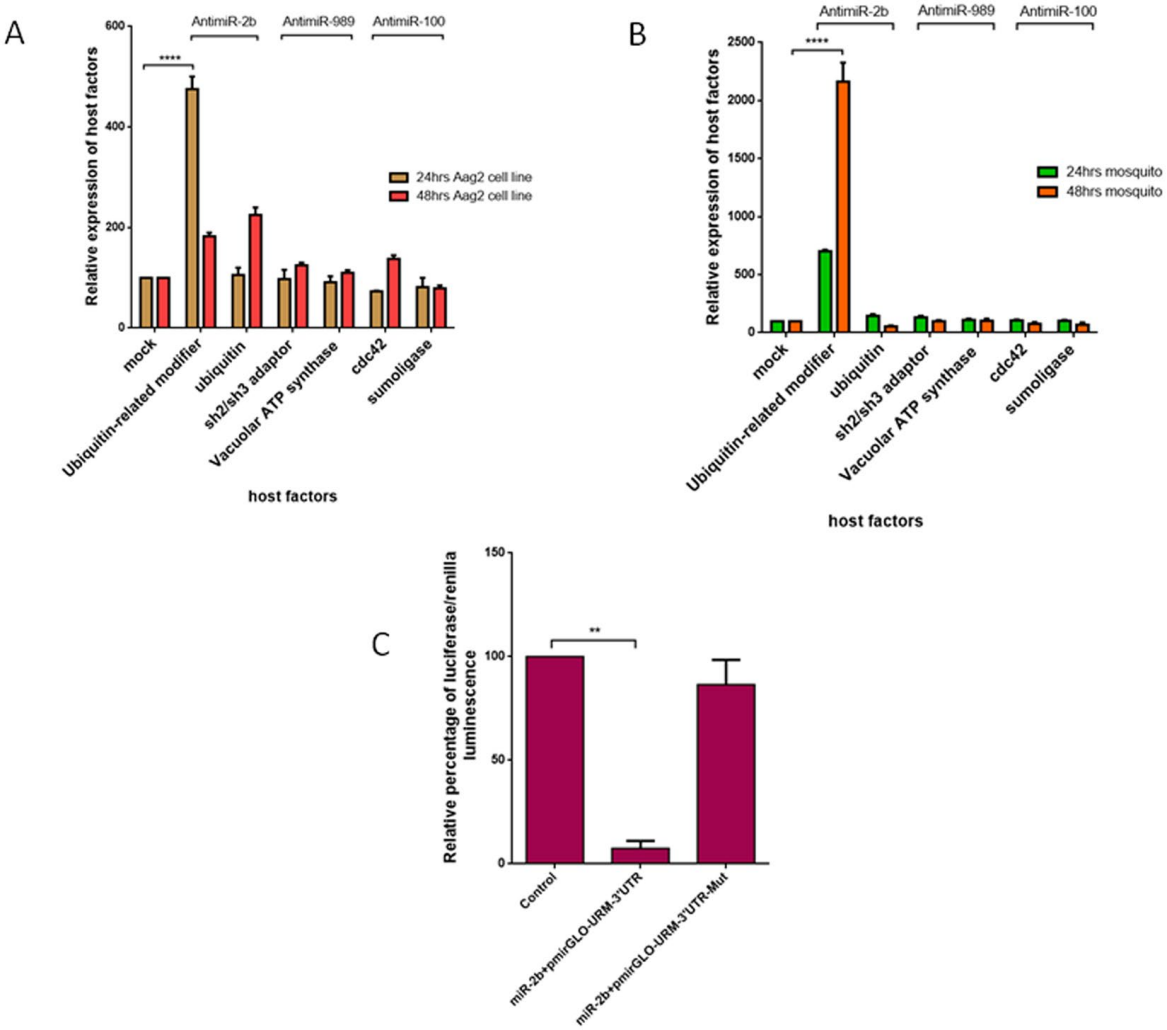
(**A**) Aag2 cell line transfected with antagomir for miR-2b, miR-989, and miR-100, 24 and 48 h after transfection, showing expression levels of ubiquitin, URM, sh2/sh3 adaptor, vacuolar ATP synthase, cdc42, and sumoligase. (**B**) Mosquito injected with antagomir for miR-2b, miR-989, and miR-100, 24 and 48 h after transfection, showing expression levels of ubiquitin, URM, sh2/sh3 adaptor, vacuolar ATP synthase, cdc42, and sumoligase. (**C**) Luciferase assay showing relative percentage of luciferase/renilla luminescence for miR-2b binding to 3′UTR of URM and to mutated 3′UTR of URM. Data were expressed as mean ± SEM except in 3 C, which is expressed in SD; ****p < 0.0001 *vs*. control group.

To understand the role of miRNAs in the expression of the putative targets, miRNAs were knocked down by either transfecting miRNA-specific antagomirs in *Ae. aegypti* cells or by injecting in the mosquitoes, and the time points that exhibit maximum silencing were ascertained **(**Supplementary Figure S2). Upon maximum knockdown of the miRNAs, the expression levels of these miRNA targets were elucidated through qRT-PCR at 24 and 48 h after miRNA silencing. Expression of URM was found to be significantly regulated by miR-2b inhibition as compared to ubiquitin at 24 h, but at 48 h, ubiquitin showed a marked increase in its expression. In the case of miR-100 and miR-989, their targets did not show significant difference in their expression levels after antagomir transfection at both 24 and 48 h (Fig. 3A). To further confirm our findings in mosquitoes, we performed loss-of-function assays of miRNAs in *Ae. aegypti* mosquitoes. The miRNA-specific antagomirs were injected into the thorax of female mosquitoes and time points similar to those for the experiment performed in the Aag2 cell line—24 and 48 h after nanoinjection—were chosen for the validation of targets identified in mosquitoes. Expression of URM was found to be significantly regulated when miR-2b was inhibited as compared to ubiquitin, whereas miR-100 and miR-989 targets did not show much difference in their expression levels **(**Fig. 3B **)**. On the basis of these findings, we hypothesized that URM is a target of miR-2b. To confirm this, we performed a luciferase assay in HEK 293-T cells to evaluate the binding of miR-2b to the 3′UTR of URM. Empty pmR-mCherry vector and pmirGLO vector containing 3′UTR of URM served as controls; sequence-specific binding of miR-2b to the 3′UTR of URM was evaluated by mutating the miRNA-binding site at the 3′UTR and then the assay was performed. The assay revealed a >80% reduction in the luciferase activity in those constructs with wild-type 3′UTR of URM. However, constructs with a mutated binding site showed loss of binding, and the luciferase activity reverted to control values. Taken together, our results establish that URM is indeed a target of miR-2b **(**Fig. 3C).

### URM affects CHIKV replication

**Figure 4:**
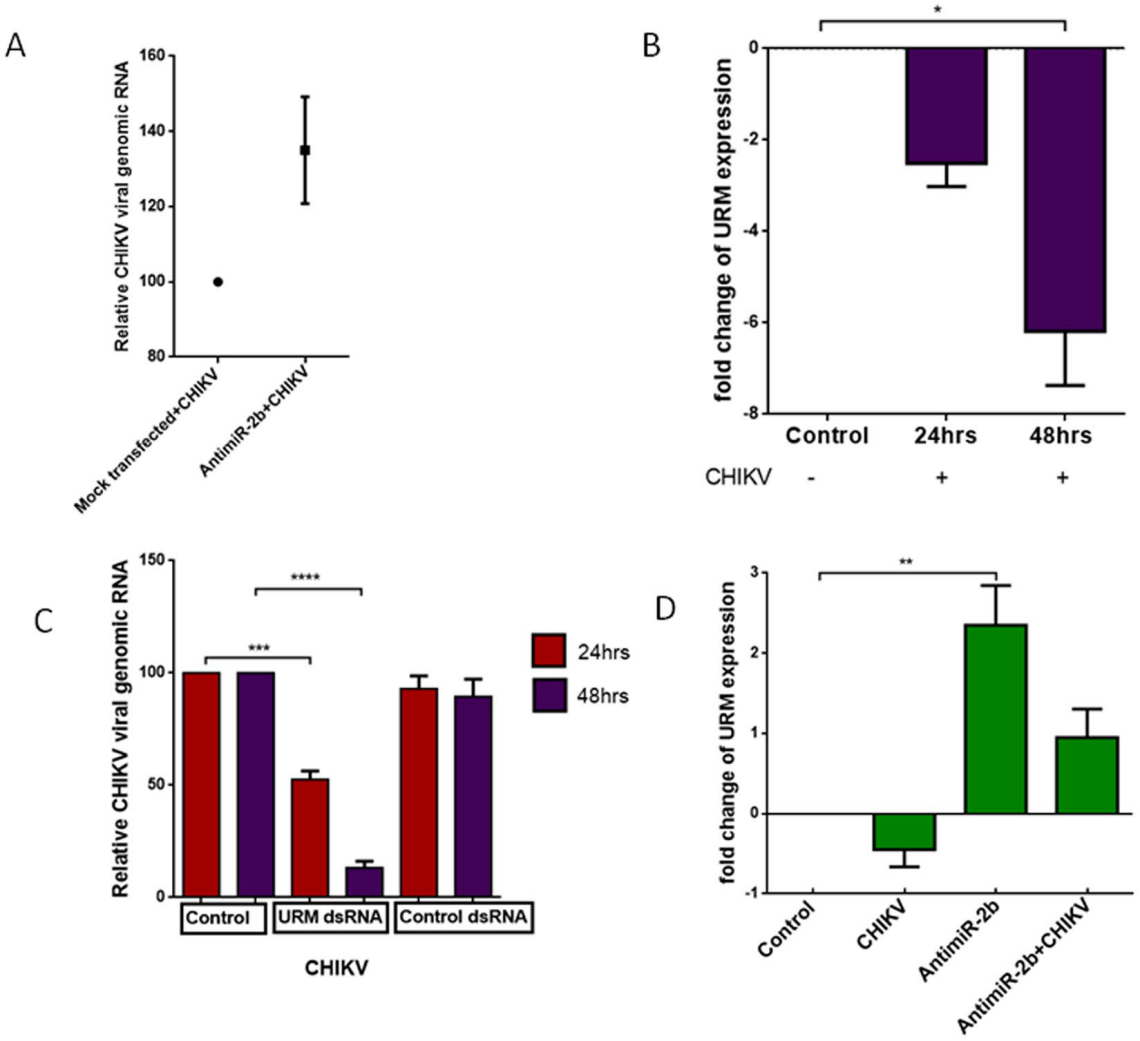
(**A**) Relative CHIKV viral genomic RNA during miR-2b inhibition (**B**) Relative expression of URM 24 and 48 h after CHIKV infection. (**C**) Relative CHIKV viral genomic RNA 24 and 48 h after infection in *Ae. aegypti* with dsRNA transfections. (**D**) Effect of miR-2b inhibitor on URM expression in CHIKV-infected and uninfected state. Data were expressed as mean ± SEM; ****p < 0.0001 *vs*. control group.

To understand the role of URM during CHIKV infection better, we dissected the expression pattern of this transcript during CHIKV infection in the presence and absence of miR-2b. As a first step, we confirmed whether silencing miR-2b regulated CHIKV infection. For this purpose, we inhibited miR-2b using antagomir in the Aag2 cell line and infected it with CHIKV virus. After 24 hours of infection, we checked the CHIKV viral genomic RNA and found that inhibiting miR-2b increased CHIKV replication **(**Fig. 4A **)**. We observed an increase in viral replication when miR-2b is silenced, thereby proving that miR-2b may be playing a role in regulating CHIKV infection in *Ae*. *aegypti*. Next, we sought to understand the role of URM during CHIKV infection, as it is a target of miR-2b. We first checked the expression of URM in *Ae. aegypti* mosquitoes 24 and 48 h after CHIKV infection. We observed more than a 2.5-fold reduction in URM expression upon CHIKV infection, which further reduced in a day-dependent manner **(**Fig. 4B). This finding prompted us to hypothesize that URM may have an impact on CHIKV replication. To test this, we evaluated CHIKV replication upon silencing URM. Following dsRNA-induced silencing of URM and subsequent infection of CHIKV in *Ae. aegypti* mosquitoes, we evaluated the CHIKV viral genomic RNA 24 and 48 h.p.i. We observed a 50% reduction (*p* < 0.001) in CHIKV viral genomic RNA in 24 h and a more drastic 87% (*p* < 0.0001) at 48 h.p.i., whereas in GFP dsRNA-transfected cells that served as the control, the CHIKV viral genomic RNA remained similar to control infection **(**Fig. 4C **)**. This finding was surprising, as we hypothesized that a reduction in URM would increase CHIKV viral genomic RNA, as the presence of virus seems to reduce the expression of URM, as can be observed in Fig. 4B. We suspected that miR-2b may be playing a role in this phenomenon and could be involved in regulating URM during CHIKV replication. To test our hypothesis, we silenced miR-2b in the mosquitoes and then tested the expression of URM upon CHIKV challenge in these mosquitoes. We observed that although there was a reduction in URM expression upon CHIKV infection, this effect was totally reversed when miR-2b was silenced; expression of URM that was downregulated upon miR-2b silencing increased by 3.5-fold (*p* = 0.005) when the miRNA-silenced mosquitoes were infected with CHIKV **(**Fig. 4D **)**.

## Discussion

*Ae. aegypti* spreads many viral diseases such as dengue and chikungunya and has more recently created global havoc with Zika transmission, causing much harm to healthcare and public health management. The most effective way of controlling these infections is through vector control. Central to vector control is the understanding of mechanisms that the vector employs to tackle the viruses it carries. Viral infection creates a competition between the vector and the virus in taking over the cellular machinery to run each of their own regime and successfully establish each of their hold. During this process, the host activates genes of several pathways to control the virus and the virus attempts to either activate the factors that help in membrane budding, which in turn will help its replication, or suppress the pathways inhibiting its replication and expansion^24,25^. The purpose of our present study was to identify the cellular factors that may play a role in viral replication. One approach is to identify the vector factors that may be regulated upon infection using several methodologies identifying host factors that regulate viral infection^26^. Another approach is to identify those regulators that regulate these factors within the cell, such as miRNAs, and then identify their targets^22^. These targets may further be validated for their functions to understand their mode of action during specific conditions. The second approach provides information at a more biological level, offering insights both into cellular transcripts and into their modulators such as miRNAs that could be regulating them in a more centralistic manner. Therefore, we utilized the second method in the present study.

An earlier report on *Ae. aegypti* had identified 86 distinct miRNAs^27^; our data expanded the list of known miRNAs to 124. Whereas the earlier study used Roche 454 platform for generating high-throughput data, our study utilized Ilumina sequencing, generating much more data, which could be a plausible reason for the identification of more number of miRNAs. The new miRNAs have already been described in earlier studies on *Ae. albopictus* ^18,28^, thereby proving that these are bonafide miRNAs.

Regulation of miRNAs during arboviral replication in *Ae. albopictus and Ae. aegypti* has been previously studied^29,30^. miRNAs regulated in these studies were found to be regulated in the present study as well. For instance, miR-285 was reported to be among the highest expressing miRNAs in the saliva of CHIKV-infected *Ae. aegypti* ^30^. Similarly, miR-317 and miR-2951-3p were found to be upregulated during dengue virus infection in *Ae*. *albopictus* ^29,31^ and miR-34-3p was shown to be involved in *Wolbachia* infection^32^. Whereas our earlier study on *Ae. albopictus* miRNAs highlighted miR-2b and miR-100 to be regulated upon CHIKV infection^18^, miR-100 has been shown to attenuate human cytomegalovirus replication via the mTOR pathway^33^ and have also been implicated in pathways related to cell cycle progression with targets such as plk1, while also playing a role in apoptosis^34^. Similarly, miR-989 has been predicted to be an important miRNA targeting insect immunity in other mosquito species as well^35,36^. The time points of our study coincided with the early replication events of CHIKV in the vector. Studies have shown that replication kinetics of the virus differ in the host and the vector, with the virus replicating at a much slower pace in the vector than in the host, probably allowing for persistent infection in the vector to enable efficient virus transmission throughout the life of the vector^9^. In addition to the several mechanisms that may be employed by the virus, host-derived factors may also play a role. Due to the significance of miRNAs in mosquito biology, we hypothesized that vector miRNAs and their targets could also be involved in regulating viral replication.

Using RNAhybrid, multiple targets for the selected miRNA were predicted, taking the seed binding region into consideration. The targets were analyzed by Gene Ontology (GO) terms and selected for further validation. Bioinformatics has been useful in identifying targets of miRNAs in the recent past^37^. Prediction tools identified a total of 8928 targets for all the regulated miRNAs taken together; on the basis of binding energies and relevant pathways, a total of six targets were chosen for the three miRNAs, to study whether the expression of these transcripts was affected by silencing the respective miRNAs that supposedly target them. Loss-of-function and gain-of-function analyses have been earlier employed to study the role of miRNAs in mosquitoes^36,38^. In the present study, out of the six transcripts, only one target, URM, showed promising results, clearly emphasizing the importance of validating miRNA targets by wet lab validations to confirm that the candidate is indeed a target of miRNA. Whereas luciferase assays provide information on binding of the miRNA to the 3′UTR of the target gene, the expression profile of the putative targets provides indirect information of the action of these miRNAs on the targets^39^.

In our study, we tested the effect of silencing of miRNAs on their putative targets in both *in vitro* conditions and mosquitoes. In the case of miR-100, of the two targets chosen (sumoligase and cdc42), cdc42 was found to be regulated in the cell line; however, we did not observe any significant regulation in the mosquito. Sumoligase expression was, however, almost negligibly regulated by the knockdown of miR-100. Cdc42 is a highly conserved small GTPase of the Rho family, acting as molecular switch in a wide range of signaling pathways such as vesicle trafficking and cell polarity^40^; sumoligase has been shown to play a role in immunity to virus infection^41^. Neuraminidase to the host cell surface is also regulated by cdc42 affecting influenza virus replication^42^. The miR-989-predicted target sh2/sh3^43^ and vacuolar ATP synthase^44^ have been found to play a role in viral infections. But sh2/sh3 adaptor and vacuolar ATP synthase were not found to be regulated when the cells or mosquitoes were transfected or injected with the respective antagomir, negating the possibility of them being a target of miR-989. Targets of miR-2b showed the most promising results. URM and ubiquitin were selected, as both of these targets have been previously reported to be significant in viral replication and infection^45,46^. URMs such as ISG15 and FAT-10 have been the shown to play a role in innate immunity and viral infection^47,48^. In our study, we found URM to be differentially regulated upon its antagomir treatment in *Ae. aegypti* cell line and *Ae. aegypti* mosquito. The expression level of URM increased 24 h after transfection with antagomir both in the cell line and in the mosquitoes. In the case of mosquitoes, there was more than 20-fold increase in its expression after 48 h of miRNA silencing, clearly emphasizing the effect miR-2b has on the target. To further validate the target of miR-2b, we performed a luciferase reporter assay that shows significant binding to the 3′UTR of URM. Most significant in our findings was the pattern of URM expression during CHIKV infection and the manner in which it was altered when miR-2b was silenced in mosquitoes.

**Figure 5:**
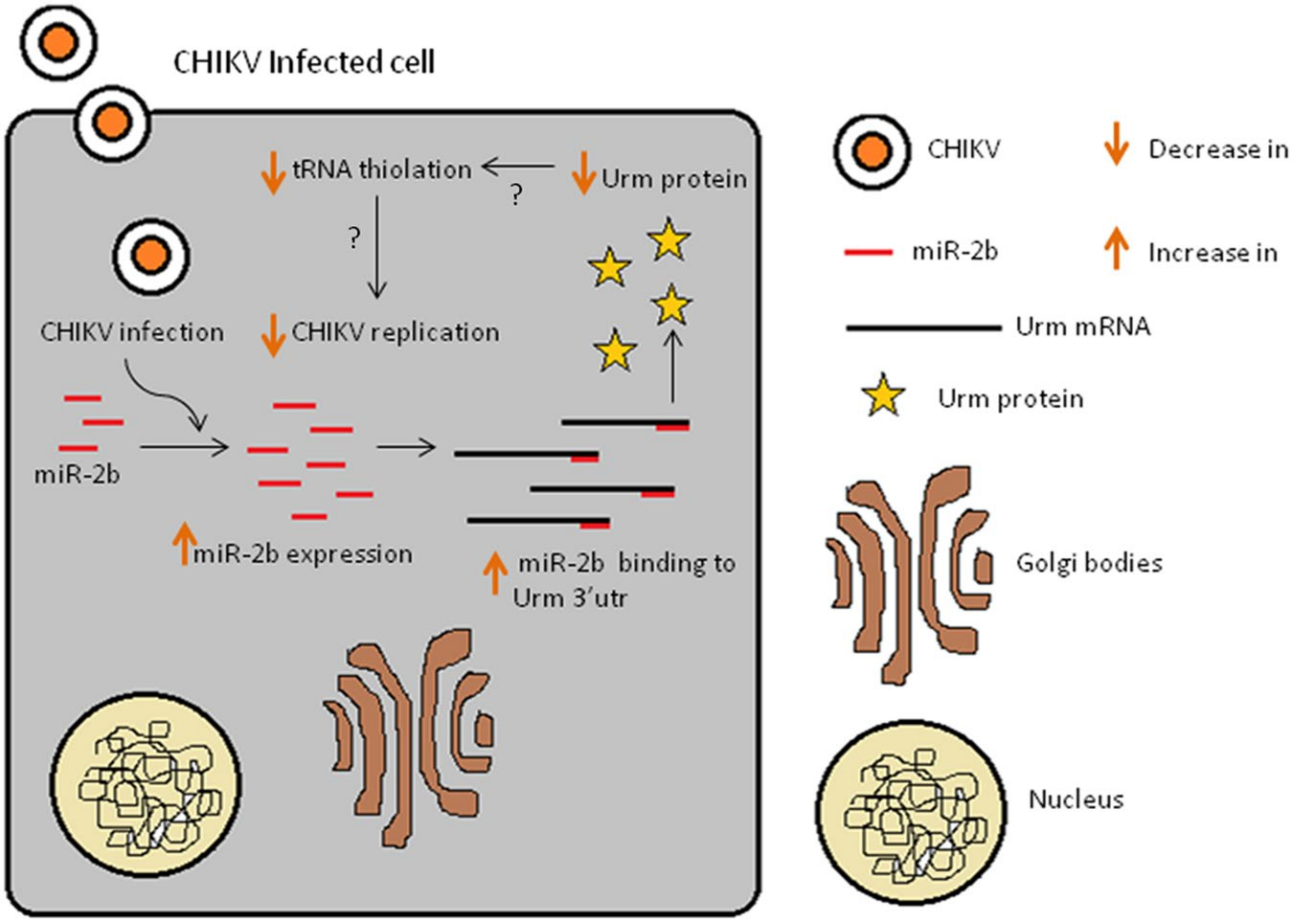
Proposed model for miR-2b affecting CHIKV replication through URM inhibition.

Based on all of our findings, we propose a model that may explain the mechanism by which CHIKV may be regulated in *Ae. aegypti* by miR-2b through its regulation of a host factor, namely, URM **(**Fig. 5). Our data reveals that miR-2b expression is upregulated during CHIKV infection in *Ae. aegypti*. Our data also reveals that miR-2b binds to URM and negatively regulates it. Several reports have implicated URM in tRNA thiolation^49,50^, which has been shown to be essential for virus replication in other systems^51^. When there is a decrease tRNA thiolation, there is a negative impact on viral replication and this is the probable mechanism by which *Ae. aegypti* may be regulating CHIKV replication. Upon CHIKV replication, *Ae. aegypti* increases the expression of miR-2b, which in turn decreases the expression of its target, URM, thereby reducing the overall tRNA thiolation in the cell. This, in turn, results in controlling CHIKV replication in the vector. The present study throws light on the regulation of 126 miRNAs in *Ae. aegypti* upon CHIKV infection. Further analysis of these miRNAs and their targets revealed that the URM transcript is an important target regulated by miR-2b controlling CHIKV replication. These findings were validated using cell lines and mosquitoes, making these results significant in studying insect immunity. The present study also provides insights into the miRNAs that could be taken up for further studies for their possible role in CHIKV replication. This study can be taken forward to investigate the possible role of URM in tRNA thiolation and its effect in CHIKV replication.

## Methods

### Cell culture and virus infection

*Ae. aegypti* cell line (Aag2) was maintained at 28 °C and 5% CO_2_ in Schneider media (Invitrogen, CA) supplemented with 10% FBS and antibiotics. The cells were infected with MOI 10 of a CHIKV isolate (accession no. JF950631.1) that was characterized during an outbreak in 2010^52^. The cells were infected in triplicates and harvested 12 and 24 h.p.i and processed further for small RNA sequencing.

### Rearing of *Ae. aegypti* mosquito and CHIKV infection

*Ae. aegypti* eggs were allowed to hatch into first instar larvae which were fed on fish food until fourth instar. Upon development, the pupae were transferred to water-filled plastic containers inside cloth cages and allowed to emerge. Upon emergence, the adult mosquitoes were reared under controlled conditions at 28 ± 2 °C, 70–75% humidity, and fed with 2% sterile glucose solution. *Ae. aegypti* mosquitoes were infected by blood-feeding using 500 μL of polyethylene glycol purified CHIKV mixed in 2 mL of rabbit blood. Previously starved mosquitoes were fed for 20 min at room temperature through membrane feeding. After blood feeding, fully fed mosquitoes were separated and taken for further processing for time point studies. All experiments related to mosquitoes and virus were performed in bio-containment facilities ACL-2 and BSL-3, respectively.

### Sample preparation for small RNA sequencing

CHIKV-infected Aag2 cells were harvested at 12 and 24 h.p.i. and stored in Trizol (Invitrogen, CA) until RNA extraction. Small RNA population from the total RNA was enriched and extracted using miRNeasy kit (Qiagen, Germany) as per manufacturer protocol. Agilent 2100 Bioanalyzer RNA Nano 6000 kit was used to check the quantity and quality of isolated RNA. Uninfected and infected samples were outsourced for small RNA sequencing by Illumina Genome Analyzer II (Agilent, CA).

### Data analysis

Data analysis was carried out using an in-house PERL-based pipeline developed for analysing small RNA data as described elsewhere^18^. The pipeline makes use of tools like Bowtie, RNAfold, and RNAplot for analysing the data^53–55^. Briefly, *Ae. aegypti* genome and coding region sequences were downloaded from VectorBase using the BioMart tool^56^. Pre-miRNA and mature miRNA sequences of arthropods were downloaded from the miRBase database V.19^57^. Other non-coding RNAs (ncRNA) sequences were fetched from the ncRNA database^58^. The downloaded sequences were indexed separately. After a quality check and adaptor trimming of small RNA reads, reads with length ≥18 nt were matched to mature miRNA sequences to identify known miRNAs. Tags per million (TPM) values were calculated for matched miRNAs. UTR sequences of *Ae. aegypti* genes were downloaded from VectorBase and targets of significant miRNAs were predicted using RNAhybrid tool^59^. The targets were filtered on the basis of complementarities of the miRNAs with the targets and energy of the miRNA:target, i.e, not exceeding −20 kcal/mol. Functional enrichment of the predicted targets was performed by WEGO webtool^60^ using GO term accession of each target gene fetched from VectorBase using the BioMart tool. The dataset analysed during the current study are available in ArrayExpress (accession number E-MTAB-5222).

### Transfection in Aag2 cell lines and CHIKV infection

Antagomirs for miR-989, miR-100, and miR-2b complementary to mature miRNA sequence were synthesized from Ambion (Life Technologies, CA). In addition, scrambled miRNA provided by the manufacturer was used as the negative control in all experiments. Antagomir transfections were performed with Attractene transfection reagent (Qiagen, Germany) in Aag2 cell line as per the manufacturer instructions. Briefly, 0.3 × 10^6^ Aag2 cells were seeded into 6-well plates to reach 75% confluency before proceeding to transfection. A volume of 100 pmol of antagomir for each miRNA, miR-2b, miR-100, and miR-989, along with the negative control was transfected and the cells were incubated at 28 °C for 4 h before changing with fresh media. The cells were collected 24, 48, and 72 h after transfection and processed for RNA isolation. Aag2 cells that were transfected with miR-2b antagomir were infected with CHIKV after 24 h and cells were collected 24 h.p.i for RNA isolation.

### Antagomir injections in mosquitoes

Female mosquitoes (4–5 days old) were divided into four batches of 100 mosquitoes each. The first batch was injected with 69 nL of negative control (scrambled miRNA). The second, third, and fourth batches of mosquitoes were injected, with 69 nL of 100 µM miR-2b, miR-100, and miR-989 antagomirs, respectively. Mosquitoes were collected at two time points, 24 and 48 h, and were stored in Trizol (Invitrogen, CA) at −80 °C until RNA extraction. Time-wise knockdown of miRNA expression following injection was checked by miRNA qRT-PCR. In the case of subsequent blood feeding studies, nanoinjected mosquitoes were allowed to revive for 24 h prior to membrane feeding. Every experiment was repeated at least three times.

### miRNAs, site-directed mutagenesis, and 3′UTR cloning

All the mature miRNAs were mapped against *Ae. aegypti* genome using Bowtie, and 250 bp flanking region from each side were extracted using in-house PERL script. miRNA miR-2b binding site 3′UTR of URM were amplified with primers containing the desired mutation for binding sites of miRNA miR-2b and cloned into the pmirGLO vector (Promega, WI) for lucifersae assay. Pre-miRNAs were PCR amplified and cloned into pmR-mCherry vector (Clontech Lab, CA) along with the 3′UTR of CHIKV which was cloned into pmirGLO vector (Promega, WI).

### Luciferase reporter assay

Luciferase activities were measured with a luminometer according to manufacturer recommendation (Glomax20/20 Luminometer, Promega, WI) 24 h after transfection using the Dual-Glo luciferase reporter assay system (Promega, WI). Renilla luciferase activity was normalized using firefly luciferase activity for each sample.

### dsRNA preparation for URM and mosquito injection

For dsRNA preparation, URM and green fluorescent protein (GFP, as control) were cloned into pGEMT-easy vector (Promega, WI) and were *in vitro* transcribed with T7 and SP6 polymerase using Promega’s Riboprobe combination kit (Promega, WI). Further dsRNAs were purified using the Trizol (Invitrogen, CA) method described earlier and stored until further use. For dsRNA-mediated silencing, 800 ng of total dsRNA was injected into a mosquito. In the case of subsequent blood-feeding studies, nanoinjected mosquitoes were allowed to recover for 24 h prior to membrane feeding. For time-series experiments, female mosquitoes were collected 24 and 48 h after feeding and stored in Trizol (Invitrogen, CA) at −80 °C until RNA extraction.

### Sample collection and RNA isolation

Antagomir-injected mosquitoes were collected 24 and 48 h after injection and Aag2 cell line 24, 48, and 72 h after transfection. RNA was isolated by the Trizol (Invitrogen, CA) method and kept at −80 °C until further use. The isolated RNA was used for qPCR reaction for the miRNA and host factor-expression profiling.

### Quantitative RT-PCR

Expression profiling was carried out by quantitative RT-PCR. For miRNAs, 1 µg of RNA was polyadenylated, reverse transcribed, and quantified by qRT-PCR using NCode miRNA First Strand cDNA Synthesis and qRT-PCR kit (Thermo Fisher Scientific, MA). qRT-PCR reactions were set up using 1:10 diluted cDNA as template following manufacturer instructions. For other transcripts, cDNA was used directly for quantitative RT-PCR using QuantiTect SYBR Green RT-PCR Kit (Qiagen, Germany). Experiments were conducted a minimum of two times, with each experiment set up in triplicates. For miRNA and transcript expression profiling, 5.8 s rRNA was used as an endogenous control. Expression levels were then calculated using the 2^−ΔΔCT^ method. Details of the qRT-PCR primers used in the study are provided in Supplementary Table S1.

### Statistical analysis

Differentially expressed significant miRNAs were predicted using edgeR package in R. The *p*-value cut-off was determined from the data, with the significance threshold selected as 0.05. Statistical analysis of experimental data was conducted using GraphPad Prism (version 5) using Student’s *t*-test when comparing two conditions. Data from different treatments were subjected to analysis of variance (ANOVA). For multiple comparisons, Dunnett’s test was performed. Values of *p* < 0.05 considered significant have been represented with an asterisk in the figures.

### Data availability

Small RNA sequencing data were deposited in ArrayExpress (accession no. E-MTAB-5222).

## Electronic supplementary material

Supplementary Information
