# Supplementary material for: Aedes aegypti microRNA miR-2b regulates ubiquitin-related modifier to control chikungunya virus replication
Source: Sci Rep. 2017 Dec 15;7:17666. doi: 10.1038/s41598-017-18043-0 (PMC5732197; doi:10.1038/s41598-017-18043-0)
Supplement: Supplementary file 1 — Supplementary Information [file 41598_2017_18043_MOESM1_ESM.pdf]

Aedes aegypti microRNA miR-2b regulates ubiquitin related modifier to control chikungunya virus replication.

Sunil Kumar Dubey[1], Jatin Shrinet[1], Jaspreet Jain[1], Shakir Ali[2], Sujatha Sunil[1]*

[1] Vector Borne Disease group, International Centre for Genetic Engineering and Biotechnology (ICGEB), Aruna Asaf Ali Marg, New Delhi, India

[2] Department of Biochemistry, Faculty of Science, Jamia Hamdard, New Delhi, India

*Corresponding author

E-mail : sujatha@icgeb.res.in (SS)

Supplementary Figure S1.

The functional analyses of the targets were carried out by Gene Ontology (GO) terms. GO terms for each of the targets were fetched from VectorBase database using BioMart tool. The terms were visualized using WEGO webtool **(Supplementary Figure S1)**. The analysis of GO terms classified the targets into three categories, namely, Biological Process, Molecular Function, and Cellular Component. Amongst Biological Processes, targets of the regulated miRNAs revealed their involvement in cell communication, cell death, cell adhesion, immune system process, metabolic process and response to external stimuli to name few. Similarly, the targets seem to function in activities like catalytic activity, protein binding, signal transducer, electron carrier and translation regulator at the Molecular Function class of GO analysis. Some of the targets were also found to be involved in activities pertaining to the cell, organelles, extracellular regions as well as synapse with respect to the Cellular Components.

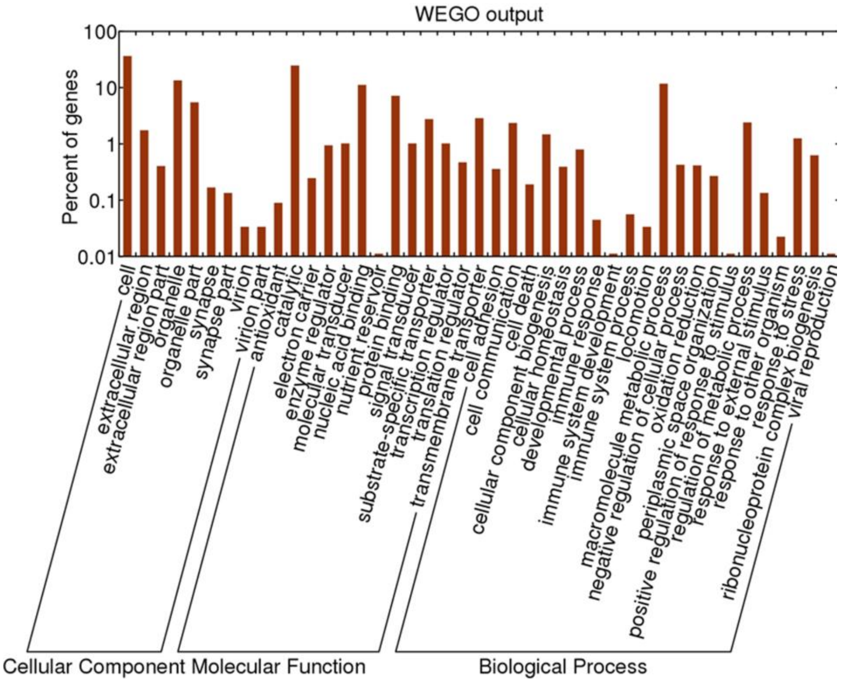

Supplementary Figure S1. The figure represents the functional classification of predicted targets of all significant miRNAs on the basis of gene ontology (GO). The targets were classified on the basis of cellular component, molecular function and Biological process.

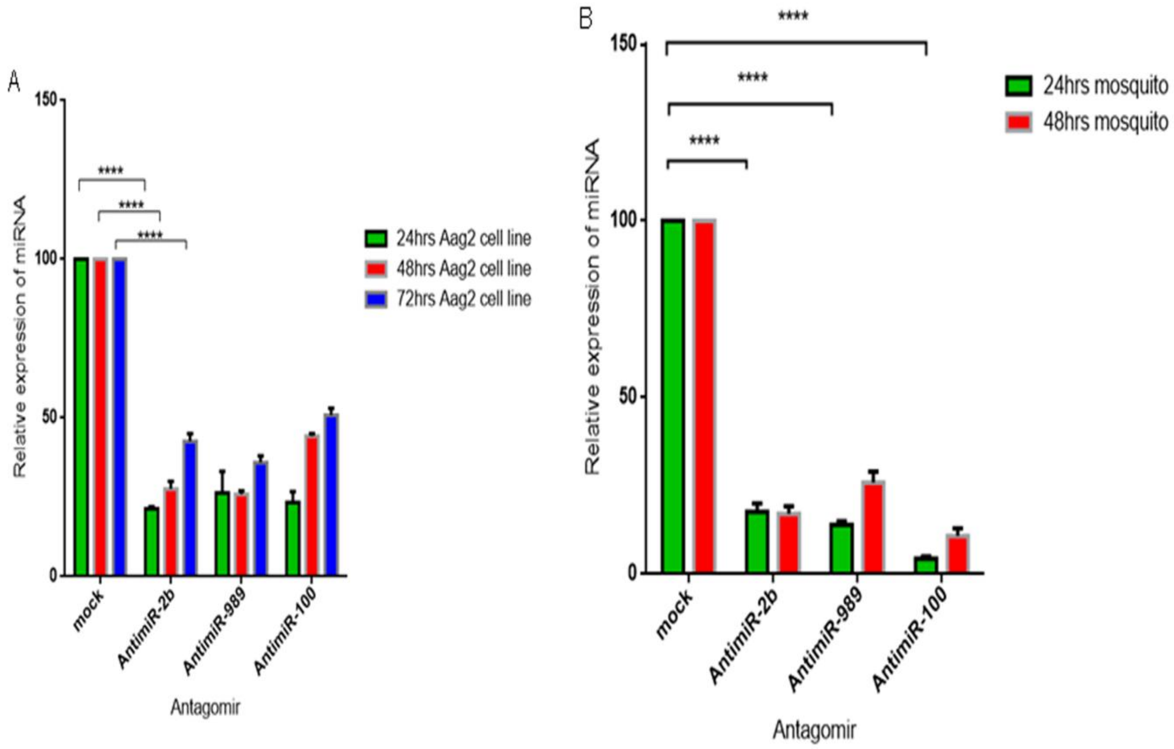

Supplementary Figure S2. MicroRNA inhibition assay. (A) To assess the inhibition level and expression of miRNAs after transfection of antagomir for miR-2b, miR-989, miR-100 into Aag2 cell line at different time poins of 24hrs, 48hrs and 72hrs post transfection. Data were expressed as mean ± SEM, ****P < 0.0001. (B) Expression level of miRNAs in *Aedes aegypti* mosquito at 24hrs and 48hrs of post antagomir injection. Data were expressed as mean ± SEM, ****P < 0.0001 vs mock group Validation of predicted targets in Aag2 cells.

Supplementary Table 1. Details of primers. The table shows the sequences of the qRT-PCR primers synthesised.

**Name**	**Fwd sequence**	**Rev sequence**
miR-2b	TATCACAGCCAGCTTTGATG	Universal
miR-100	AACCCGTAGATCCGAACTTGTG	Universal
miR-989	TGTGATGTGACGTAGTGGTAC	Universal
AAEL008680	ACGACACCGACTGGGATCTA	GCCAAGCAACAAGCAGGAAA
AAEL006511	CGGAAGAGACACGTTGGGAA	CAGCACCAGATGGAGAGTGG
AAEL011500	CAGTGTTAGCGTCCTGGTCA	CGACTGTGCCATCTCCTACC
AAEL015099	AGCGGGGTTTCAGCTATGTC	TCGGATGGAATGCCAAACCT
AAEL013539	TACTGAACACTCACGGCCAC	GGTGGTCCTTCTTAGGTGCC
AAEL012819	CGTCTAAAGCAGGCCAAGGA	TCCGGTGGTCCCAGTTAGAT
5.8s	CCCTAGGCAGGGGATCACT	GGCACTCAAGAATGTGTGCAT

Details of small RNA analysis

The reads of Uninfected, 12 h.p.i. and 24 h.p.i. libraries were processed using in-house pipeline. Reads were trimmed and mapped to downloaded miRNAs and other non-coding RNAs. Small RNA processing results are shown in supplementary Table 2.

Supplementary Table 2. Table represents the distribution of small RNA reads of all the three libraries.

S.No	Sample	Raw reads	Reads mapped to known miRNAs	Total miRNA identified	tRNA	rRNA	snoRNA; CD-box
1.	Uninfected	1.07×10^{7}	3.74×10^{5}	116	4.70×10^{5}	1.60×10^{6}	8.90×10^{5}
2.	12 h.p.i.	2.97×10^{7}	1.34×10^{6}	124	1.54×10^{6}	5.30×10^{6}	2.84×10^{6}
3.	24 h.p.i.	1.26×10^{7}	6.82×10^{5}	111	2.43×10^{6}	6.06×10^{6}	2.89×10^{6}

A total of 5.30×10^{7} reads were present, out of which 1.07×10^{7} reads were found in uninfected library, 2.97×10^{7} reads were present in 12 h.p.i. library and 1.26×10^{7} reads were present in 24 h.p.i. library. A total of 126 known miRNAs were identified in all the libraries taken together, out of which 116 miRNAs were identified in uninfected library whereas 124 and 111 known miRNAs were identified in 12 h.p.i. and 24 h.p.i. libraries respectively (Supplementary Table 3). Out of total 126 miRNAs identified in all the libraries, 106 miRNAs were present in all the three libraries, nine miRNAs namely, miR-2944b-5p, miR-927, miR-2943, miR-281-3p, miR-2944a-3p, miR-316, miR-2944b-3p, miR-309b-3p and miR-2944a-5p were found in both uninfected and in 12 h.p.i. library whereas four miRNAs namely, miR-1175-5p, miR-iab-4-5p, miR-2796 and miR-125-3p were found in both 12 h.p.i. and 24 h.p.i. library. miR-310-5p was identified only in uninfected library and miR-1 was present only in 24 h.p.i. library. Five miRNAs namely, miR-3425, miR-2755-3p, miR-279-5p, miR-981 and miR-929-5p were found to be present only in 12 h.p.i. library.

The identified miRNAs were further classified into four categories based on the abundance of reads as inferred by TPM values of the miRNAs. The categories were (i) significantly abundant (SA) with

TPM ≥ 1000 (ii) moderately abundant (MA) with TPM between 100-999 (iii) abundant (A) with TPM between 10-99 and (iv) rare (R) with TPM value ≤ 10. The analysis revealed that eight miRNAs of uninfected library were in SA category whereas twelve and eleven miRNAs of 12 h.p.i. and 24 h.p.i. libraries respectively were in SA categories. MA categories consist of twenty three, twenty two and sixteen miRNAs from uninfected, 12 h.p.i. and 24 h.p.i. libraries respectively. Twenty one, twenty three and twenty six miRNAs of uninfected, 12 h.p.i. and 24 h.p.i. libraries respectively were in A category. Most of the miRNAs from all three libraries were found to be present in R category. 64 miRNAs from uninfected library, 67 miRNAs of 12 h.p.i. and 58 miRNAs from 24 h.p.i. libraries were present in rare category. miR-184 was found to be most abundant microRNA present in all the three libraries.

Supplementary Table 3. Details of miRNAs. The table shows all the miRNAs identified in the study. TPM values of each of the miRNAs are shown in the table. Symbol "-" represents that the miRNA was not identified in the respective library.

miRNA	Uninfected	12h.p.i.	24h.p.i.
miR-2b	52.57161	350.4825	230.2844
miR-989	243.7834	329.3553	86.9867
miR-317-5p	12.09612	14.56699	65.79556
miR-71-5p	15.63191	23.95311	58.8531
miR-998-3p	150.4572	274.6197	54.75074
miR-34-3p	6.885486	9.688899	40.1558
miR-278-5p	5.76892	7.19939	30.13657
miR-2951-3p	251.2272	289.3213	13.33267
miR-100	172.4163	288.6821	9.814746
miR-10-5p	1.302659	15.17255	7.810264
miR-2951-5p	6.327183	0.067284	1.89302
miR-2779	21.02865	17.79662	1.262265
miR-285	6.699391	8.645997	0.552241
miR-310-5p	0.093047	-	-
miR-279-5p	-	0.33642	-
miR-929-5p	-	0.168202	-
miR-981	-	0.067284	-
miR-3425	-	0.06728	-
miR-2755-3p	-	0.033642	-
miR-316	0.744377	1.143828	-
miR-927	0.744377	1.00926	-
miR-2943	0.465236	0.706482	-
miR-2944a-5p	0.837424	0.605556	-

miR-2944b-3p	0.744377	0.50463	-
miR-281-3p	0.093047	0.134568	-
miR-2944a-3p	0.186094	0.100926	-
miR-2944b-5p	0.093047	0.100926	-
miR-309b-3p	0.279141	0.033642	-
miR-184	8398.99	11179.85	18219.14
miR-2940-3p	4491.942	5003.105	7299.836
bantam-3p	3241.761	3796.837	5689.58
miR-283	2809.743	3059.976	3803.914
miR-11-3p	1545.792	2155.948	2166.52
miR-276-3p	2244.017	3002.079	2159.42
miR-306-5p	988.7185	1331.921	1899.393
miR-14	1248.878	1663.463	1836.122
miR-2940-5p	1180.302	1619.863	1187.712
miR-275-3p	955.8729	1515.001	1041.29
miR-281-5p	601.2704	818.0391	1035.452
miR-317-3p	851.6601	1045.022	836.566
miR-71-3p	358.4174	476.0681	681.623
miR-12-5p	230.4777	274.7543	668.527
miR-2a-3p	457.9778	512.166	614.8019
miR-92b-3p	474.7263	669.8124	578.9851
miR-305-5p	718.5097	1022.448	573.226
miR-8-3p	516.1323	557.6163	549.4797
miR-13-3p	450.5341	642.7979	462.6201
miR-996	151.2946	194.1817	206.1436
miR-279-3p	176.5104	262.7441	195.9666
miR-1889-5p	359.8131	627.7599	191.6276
miR-970-3p	136.5001	219.9515	183.5806
miR-282-5p	198.9347	290.6333	171.1947
miR-252-5p	133.8948	186.0403	137.5869
miR-9c-5p	187.3969	196.1329	109.2648
miR-12-3p	24.93662	37.91455	74.31584
miR-87	101.1422	158.7567	70.68683
miR-9c-3p	92.30273	125.4511	61.92987
miR-34-5p	34.33438	52.04419	53.40958
miR-277-3p	42.42948	48.04079	47.17715
miR-92a-3p	78.25261	131.8767	46.94048
miR-305-3p	40.2894	66.03927	44.65262
miR-278-3p	38.42845	56.2158	36.21122
miR-190-5p	25.30881	30.14324	36.13233
miR-2765	32.0082	52.68339	27.61204
miR-9b	54.33951	60.08463	21.61629
miR-79-3p	30.70554	47.73801	20.90626
miR-33	16.37629	20.08428	20.90626

miR-1889-3p	9.118616	8.545071	17.98727
miR-988-3p	8.65338	10.76544	17.35614
miR-13-5p	18.23723	21.76638	16.17277
miR-2945-3p	11.91003	25.56793	15.38385
miR-8-5p	6.978533	12.24569	15.30496
miR-308-3p	6.606344	5.550932	11.36038
bantam-5p	2.698366	2.859571	8.756963
miR-308-5p	1.860942	2.321299	7.415806
miR-1890	7.164627	8.982417	5.6013
let-7-5p	10.70042	18.43582	4.891276
miR-306-3p	0.372188	0.302778	4.496819
miR-965	7.07158	10.3281	3.55012
miR-282-3p	4.931496	5.920994	3.313445
miR-11-5p	2.791413	3.566053	3.313445
miR-276-5p	2.605319	3.633337	2.997879
miR-275-5p	12.74745	24.12132	2.761204
miR-2941	5.489779	5.51729	2.761204
miR-2946	3.907978	4.709882	2.366747
miR-137-3p	4.094072	10.05896	2.208964
miR-210-3p	1.209612	2.893213	2.130072
miR-1175-3p	2.512272	6.627476	2.05118
miR-998-5p	2.419225	3.162349	1.7356
miR-252-3p	0.744377	1.547533	1.49894
miR-263a-3p	0.744377	1.17747	1.49894
miR-92a-5p	1.581801	1.850311	1.341156
miR-988-5p	0.279141	0.437346	1.183373
miR-932-5p	6.234156	8.679639	1.02559
miR-957	0.372188	1.042902	0.946699
miR-133	0.279141	0.67284	0.631132
miR-1000	0.558283	0.639198	0.631132
miR-277-5p	0.279141	0.134568	0.631132
miR-375	4.18712	6.963896	0.552241
miR-92b-5p	1.860942	2.354941	0.473349
miR-980-3p	2.419225	2.153089	0.473349
miR-125-5p	1.116565	1.412964	0.394458
miR-190-3p	0.744377	0.975618	0.394458
miR-79-5p	0.65133	0.740124	0.394458
miR-2945-5p	0.279141	0.235494	0.394458
miR-980-5p	2.23313	2.893213	0.236675
miR-2a-5p	0.372188	0.908334	0.236675
miR-9a	0.558283	0.571914	0.236675
miR-1175-5p	-	0.4037	0.236675
miR-1891	0.186094	0.16821	0.236675
miR-263a-5p	0.093047	0.16821	0.157784

miR-315-5p	1.023518	1.110186	0.157783
miR-2942	1.581801	1.076544	0.157783
miR-219	0.558283	0.403704	0.157783
miR-210-5p	0.279141	0.100926	0.157783
miR-1	-	-	0.078892
miR-307	1.488754	2.220373	0.078892
miR-263b-5p	0.558283	0.84105	0.078892
miR-2c-3p	5.675873	0.740124	0.078892
miR-1174	0.465236	0.403704	0.078892
miR-2796	-	0.33642	0.078892
miR-286b	0.186094	0.269136	0.078892
miR-31	0.65133	0.235494	0.078892
miR-286a	0.186094	0.201852	0.078892
miR-125-3p	-	0.0672	0.078892
miR-iab-4-5p	-	0.033642	0.078892
